# Laser assisted synthesis of anisotropic metal nanocrystals and strong light-matter coupling in decahedral bimetallic nanocrystals†

**DOI:** 10.1039/d0na00829j

**Published:** 2021-01-19

**Authors:** Fadime Mert Balci, Sema Sarisozen, Nahit Polat, C. Meric Guvenc, Ugur Karadeniz, Ayhan Tertemiz, Sinan Balci

**Affiliations:** Department of Photonics, Izmir Institute of Technology 35430 Izmir Turkey sinanbalci@iyte.edu.tr; Department of Chemistry, Izmir Institute of Technology 35430 Izmir Turkey; Department of Materials Science and Engineering, Izmir Institute of Technology 35430 Izmir Turkey; Department of Physics, Izmir Institute of Technology 35430 Izmir Turkey

## Abstract

The advances in colloid chemistry and nanofabrication allowed us to synthesize noble monometallic and bimetallic nanocrystals with tunable optical properties in the visible and near infrared region of the electromagnetic spectrum. In the strong coupling regime, surface plasmon polaritons (SPPs) of metal nanoparticles interact with excitons of quantum dots or organic dyes and plasmon–exciton hybrid states called plexcitons are formed. Until now, various shaped metal nanoparticles such as nanorods, core–shell nanoparticles, hollow nanoparticles, nanoprisms, nanodisks, nanorings, and nanobipyramids have been synthesized to generate plasmon–exciton mixed states. However, in order to boost plasmon–exciton interaction at nanoscale dimensions and expand the application of plexcitonic nanocrystals in a variety of fields such as solar cells, light emitting diodes, and nanolasers, new plexcitonic nanocrystals with outstanding optical and chemical properties remain a key goal and challenge. Here we report laser-assisted synthesis of decahedral shaped noble metal nanocrystals, tuning optical properties of the decahedral shaped nanocrystals by galvanic replacement reactions, colloidal synthesis of bimetallic decahedral shaped plexcitonic nanocrystals, and strong plasmon–plasmon interaction in bimetallic decahedral shaped noble metal nanocrystals near a metal film. We photochemically synthesize decahedral Ag nanoparticles from spherical silver nanoparticles by using a 488 nm laser. The laser assisted synthesis of silver nanoparticles yields decahedral (bicolored) and prism (monocolored) shaped silver nanocrystals. The decahedral shaped nanoparticles were selectively separated from prism shaped nanoparticles by centrifugation. The optical properties of decahedral nanocrystals were tuned by the galvanic replacement reaction between gold ions and silver atoms. Excitons of J-aggregate dyes and SPPs of decahedral bimetallic nanoparticles strongly couple and hence decahedral shaped plexcitonic nanoparticles are prepared. In addition, localized SPPs of decahedral shaped bimetallic nanocrystals interact strongly with the propagating SPPs of a flat silver film and hence new hybrid plasmonic modes (plasmonic nanocavities) are generated. The experimental results are further fully corroborated by theoretical calculations including decahedral shaped plexcitonic nanoparticles and decahedral nanoparticles coupled to flat metal films.

## Abstract 2

The advances in colloid chemistry and nanofabrication allowed us to synthesize noble monometallic and bimetallic nanocrystals with tunable optical properties in the visible and near infrared region of the electromagnetic spectrum.

## Graphical abstract

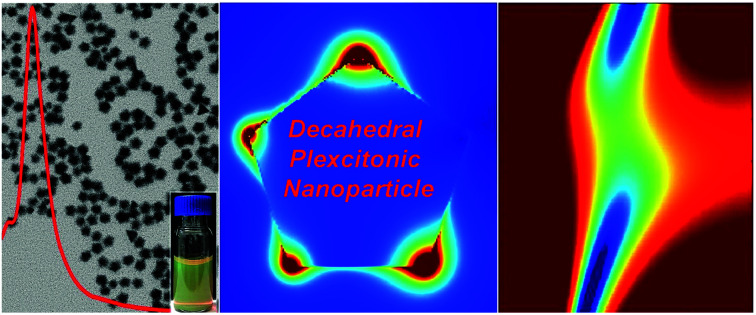

## Introduction

Noble metal nanoparticles (NPs), especially of gold and silver, have been extensively studied over several years by many authors owing to their outstanding chemical and physical properties.^1^ The optical properties of noble metal NPs supporting localized surface plasmon polaritons (SPPs) have attracted a great amount of interest, and they have found applications in label-free sensors,^2^ and surface enhanced spectroscopies.^3,4^ In fact, in the strong coupling regime, the incident electromagnetic field (photons) interacts strongly with collective electron charge oscillations on the metal surface (plasmons) and SPPs (polaritons) are generated. The SPPs on the metal nanoparticle surface are confined to a very small subwavelength region and hence they are localized. Actually, the noble metal nanoparticles serve as nanoscale antennas that enable strong localization and guidance of optical fields at nanoscale dimensions.^5^ Further, when the distance between the metallic nanostructures is in the nanometer range, *i.e.* smaller than around 10 nm, plasmonic nanocavities supporting new hybrid plasmonic modes with small mode volume and extremely large local electric field intensity are created.^6–8^ The strong interaction is resonant at a frequency that strongly depends on the size, shape, composition (monometallic or bimetallic), and dielectric constant of the surrounding environment of the metal nanoparticles.^9^ Although the optical properties of metal NPs slightly change with the size of the NPs, the shape anisotropy enormously modifies the plasmonic properties of metal NPs. Therefore, until now, metallic nanostructures with various shapes such as nanocubes,^10^ nanoprisms,^11^ nanorods,^12^ nanodisks,^13^ nanostars,^14^ nanorings^7^ have been synthesized through a variety of chemical and physical methods. The colloidal synthesis of metal NPs in aqueous medium has aroused a lot of interest since the optical and chemical properties of metal nanoparticles are easily tunable, and it is scalable, cost effective, and practical.^15^ In general, the colloidal synthesis of metal NPs consists of metal ions, complexing agents, weak and strong reducing agents, and, consequently, the corresponding colloidal synthesis reaction is usually triggered and driven by heat, light, or, in some cases, a reducing agent. For example, in photoinduced methods, visible and ultraviolet light sources have been used to convert isotropic shaped metal nanoparticles into anisotropic shaped metal nanoparticles.^16^ Mirkin and coworkers converted spherical silver NPs into silver nanoprisms by dual-beam illumination of silver NPs.^17^ In another example, monodisperse decahedral shaped silver NPs were synthesized in high yield by photochemical transformation of spherical silver NPs in water.^16^ Bimetallic nanoparticles are of more interest than monometallic nanoparticles as they show better physical, chemical, and catalytic properties.^7^ For example, Mirkin *et al.* reported gold- and silver-alloyed triangular nanoframes synthesized by etching silver nanoprisms with HAuCl_4_.^18^ Furthermore, another study reported that Au–Ag bimetallic nanoparticles showed very high photothermal stability.^19^ Recently, we have demonstrated plasmonic nanocavities in bimetallic nanorings, which have empty space that is accessible to a variety of molecules, ions, and quantum dots.^7^

**Fig. 1:**
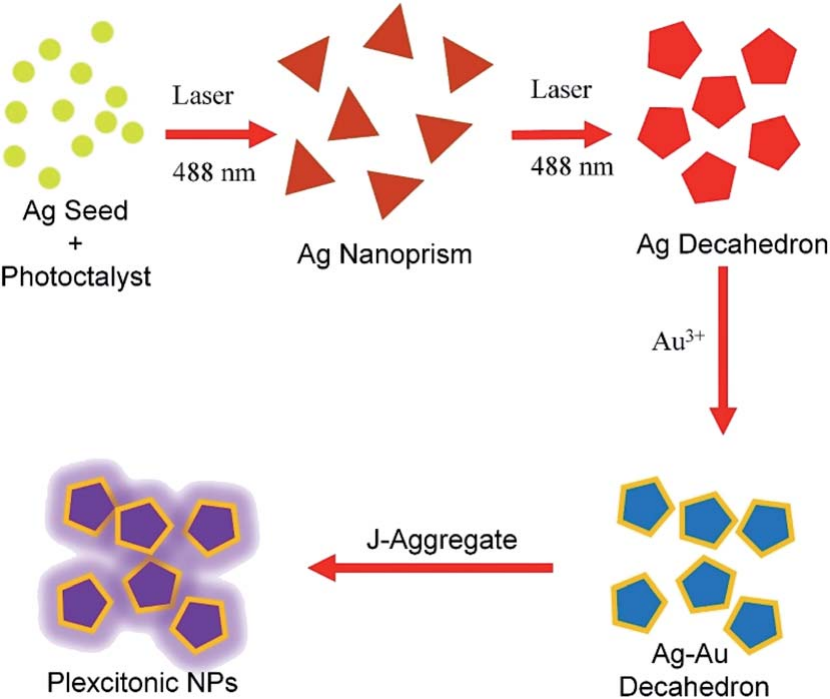
Laser assisted synthesis of decahedral Ag nanocrystals from spherical Ag nanocrystals. The scheme depicts the synthesis of colloidal decahedral shaped plexcitonic nanoparticles. Chemically synthesized spherical Ag NPs in water are irradiated at room temperature with a blue laser at 488 nm. Ag NPs that appear yellow to the naked eye are first converted to triangular shaped Ag nanoprisms (monocolored) and then to Ag decahedra (bicolored). Owing to the galvanic replacement reaction between gold ions and silver atoms on the surface of decahedral silver NPs, adding gold ions into the colloid tunes the plasmon resonance wavelength of the colloid. Excitons of the J-aggregate dye and localized plasmons of decahedral bimetallic NPs strongly interact and hence decahedral shaped plexcitonic nanoparticles are generated in the strong coupling regime.

In the strong coupling regime, metal NPs interact strongly with a molecular transition or electron–hole pairs, namely excitons, and two new hybrid modes (plexcitons) are formed.^20,21^ In this regime, the exchange rate of energy between plasmons and excitons exceeds their individual dephasing rates and hence the coupled system shows new hybrid eigenstates, which are half-plasmonic and half-excitonic.^22^ At zero detuning, the energy separation between the upper and lower polariton branches representing the strength of the coupling is called Rabi splitting energy, ℏ*Ω* = 2*g* where *g* stands for the coupling constant indicating the degree of strong coupling. Until now, many studies on plasmon–exciton hybrid nanoparticles focused on plasmonic nanostructures with a variety of shapes,^23^*e.g.*, core–shell nanoparticles,^20^ nanoprisms, hollow gold nanoprism,^24^ nanorods, nanoparticle dimers,^25^ nanorings,^7^ and recently nanodisks.^13^ However, in order to boost plasmon–exciton interaction at nanoscale dimensions and expand the application of plexcitonic nanoparticles in a variety of fields such as solar cells,^26^ light emitting diodes, and nanolasers, new plexcitonic nanoparticles with outstanding optical and chemical properties remain a key goal and challenge. The new results in this direction will promote our understanding of plasmon–exciton coupling and pave the way towards active hybrid plasmonic devices at nanoscale dimensions. Here we report colloidal synthesis of bimetallic decahedral shaped plexcitonic nanoparticles with tunable optical properties, see Fig. 1. Different from the previous studies, the present work contains the following advantages: (i) laser assisted synthesis of colloidal decahedral shaped plasmonic nanoparticles, (ii) selective separation of nanodecahedra (bicolored) from nanoprisms (monocolored), (iii) colloidal synthesis of bimetallic plasmonic nanodecahedra with tunable optical properties, (iv) colloidal decahedral shaped plexcitonic nanoparticles, (v) plasmon–plasmon hybridization (plasmonic nanocavity formation) between decahedral shaped bimetallic nanoparticles and flat metal film. Besides, all of the experimental results are fully corroborated by theoretical calculations.

## Experimental section

### Chemicals

l-Arginine, polyvinylpyrrolidone (PVP) (40000), NaBH_4_, AgNO_3_, trisodium citrate, HAuCl_4_, were all purchased from Sigma Aldrich and used without any further purification. 5,5′,6,6′-Tetrachlorodi(4-sulfobutyl)benzimidazolocarbocyanine (TDBC) dye showing J-aggregate properties at high concentration in aqueous medium was purchased from FEW Chemicals GmbH. Milli-Q water with a resistivity of 18.2 MΩ cm was used in all of the experiments.

### Synthesis of Ag seeds and laser assisted synthesis of decahedral nanoparticles

Spherical Ag NPs were used as seeds in the laser assisted synthesis of anisotropic decahedral Ag NPs, see Fig. 1 for a schematic representation of the laser assisted synthesis of nanocrystals. 0.5 ml of 50 mM sodium citrate was loaded in a glass vial containing 7 ml of water and, subsequently, 15 μl of 50 mM PVP was added. As a source of silver, 200 μl of 5 mM AgNO_3_ was added. Silver ions were reduced by adding 80 μl of 100 mM NaBH_4_, a strong reducing agent. The spherical Ag NPs were synthesized by stirring the aqueous solution for several minutes. In fact, the appearance of a bright yellow colored solution is a very strong indication of Ag nanoparticle formation. These nanoparticles show localized SPP resonance wavelength at around 400 nm. In the final step, 50 μl of 5 mM l-arginine was quickly injected into this solution before the laser irradiation of the spherical Ag colloid. In order to synthesize decahedral shaped Ag NPs, the bright yellow colored Ag colloid was exposed to a 488 nm wavelength blue laser (50 mW, CrystaLaser) for several hours, ∼15 hours. It is noteworthy that the shape transformation of the colloid was performed under vigorous stirring at room temperature. In addition, after the synthesis was complete, the colloid was centrifuged at 5000 rpm for 15 minutes in order to remove unreacted reaction precursors, and reaction byproducts.

### Synthesis of Ag–Au alloyed nanoparticles

In order to synthesize bimetallic decahedral NPs, first, the Ag decahedral colloid was synthesized, Fig. 1. After the reaction, 4 ml of the synthesized colloid was centrifuged two times at 5000 rpm for 15 minutes and then the pellet was dispersed in 1 ml water. Afterwards, 100 μl of 50 mM trisodium citrate was added and 50 μl of 0.05 mM HAuCl_4_ solution was then added dropwise into the Ag nanodecahedral colloid several times for obtaining Ag–Au alloyed NPs. Owing to the galvanic exchange of silver atoms with gold ions, the silver decahedral nanoparticles were coated with gold. Therefore, the stability of bimetallic nanoparticles is significantly improved as compared to the monometallic Ag nanoparticles due to the stability of gold. Increasing the total amount of HAuCl_4_ injected into the Ag decahedral colloid resulted in coating of decahedral nanoparticles. Further, Ag–Au alloyed nanoparticles were centrifuged at 15000 rpm for 10 minutes. After the centrifugation was complete, the supernatant was discarded, and the precipitate was then redispersed in water.

### Synthesis of plexcitonic decahedral NPs

Plexcitonic decahedral shaped NPs were prepared by mixing 1 ml of the alloyed decahedral colloid with varying amounts of 0.1 mM TDBC dye, see Fig. 1. The mixture was overnight incubated in the dark. Thereafter, the obtained colloid was centrifuged at 15000 rpm for 10 minutes. In order to remove all of the uncoupled dye molecules from the colloid, the supernatant was discarded, and the precipitate was dispersed in water again. Therefore, the decahedral shaped plexcitonic NPs shown in this study do not contain any uncoupled dye molecules or bare plasmonic nanoparticles in the colloid.

### Characterization

In order to investigate morphological evolution of the nanocrystals, Scanning Transmission Electron Microscopy (STEM) (SEM; Quanta 250, FEI, Hillsboro, OR, USA) was used. The samples were prepared by drop-casting nanocrystal suspensions onto a 200 mesh carbon-coated copper grid. Extinction measurements of the colloids in a 1 cm quartz cuvette were performed by using a balanced deuterium–tungsten halogen light source (DH2000-BAL, Ocean Optics), and a fiber coupled spectrometer (USB4000, Ocean Optics). All of the characterization measurements were carried out at room temperature.

### Numerical calculations

The finite difference time domain (FDTD) method was applied to investigate the optical properties of bare and coupled plasmonic nanoparticles, and plasmon–plasmon hybridization in decahedral nanoparticles on flat Ag films. The size of the decahedral shaped nanoparticles was indeed deduced from the STEM images. In FDTD simulations, the plane wave moves in the *z*-axis. The mesh size is 1 nm during the extinction spectra simulations and 0.1 nm during the electric field map simulations. The electric field polarization is varied and the resulting electric field distribution for each electric field polarization is then obtained in a heat map, see the ESI.† In the numerical calculations, the decahedral shaped metallic nanoparticle was suspended in the air. The localized Frenkel exciton of J-aggregates was assumed to be a Lorentzian lineshape. A very narrow dip in the extinction spectra is indeed a very clear indication of strong coupling between an exciton and a plasmon. FDTD simulation of plasmon–plasmon coupling was investigated in the Kretschmann configuration. A prism was used to couple incident light to surface plasmons of the metal film. The localized SPPs of decahedral nanoparticles were assumed to be Lorentzian and expressed as *ε*(*ω*) = *ε*_∞_ + *f*_0_(*ω*_0_^2^/(*ω*_0_^2^ − *ω*^2^ − i*γ*_0_*ω*)) where the resonance wavelength of the oscillator was set to 579 nm (2.14 eV) and the width of the plasmon resonance (*γ*_0_) was set to around 122 meV. The background index, *ε*_∞_, was set to 2.1. Theoretical dispersion curves were obtained by acquiring the reflection spectra for each incidence angle within a broad wavelength range and then the resulting reflectivity distribution for each incidence angle was obtained in a heat map, see the ESI.†

### Plasmon–plasmon coupling

In order to study plasmon–plasmon coupling in decahedral nanoparticles on flat metal surfaces, the well-known Kretschmann configuration was used.^27^ The Kretschmann configuration is commonly used for the excitation of surface plasmons on thin metal films.^21^ Flat silver films on a glass substrate were grown by thermal evaporation of Ag under vacuum. The thickness of the Ag film is 40 nm and the purity of the Ag is 99.9%. Glass substrates were cleaned with a piranha solution, a 3 : 1 mixture of sulfuric acid (95%) with hydrogen peroxide (30%). The dispersion curves were generated by using a tunable laser light source with a spectral width of around 1 nm; *i.e.*, supercontinuum laser (Koheras-SuperK Versa) with an acousto-optic tunable filter working in the visible and near infrared region of the electromagnetic spectrum. A glass prism made of BK7 was used to couple incident light to surface plasmons on the flat Ag film. An index matching fluid was used to provide the index continuity between the glass substrate and the right angle glass prism. Therefore, surface plasmons of the flat silver film and photons of the incident light strongly couple and hence surface plasmon polaritons are generated on the flat metal films. Freshly prepared Ag thin films were dipped into 10 ml of 10 mM 16-mercaptohexadecanoic acid (90%) in isopropanol for 30 minutes and then the substrates were washed with ample amount of isopropanol. Afterwards, the surface was further modified with 30 mM (3-aminopropyl)trimethoxysilane (97%) in isopropanol for 30 minutes and then the substrates were washed with ample amount of isopropanol and then water. Finally, the decahedral bimetallic colloid was placed on top of the modified Ag film for 30 minutes and then washed with ample amount of water. Subsequently, the coupled decahedral bimetallic nanoparticle-film structure was used for optical characterization.

## Results and discussion

**Fig. 2:**
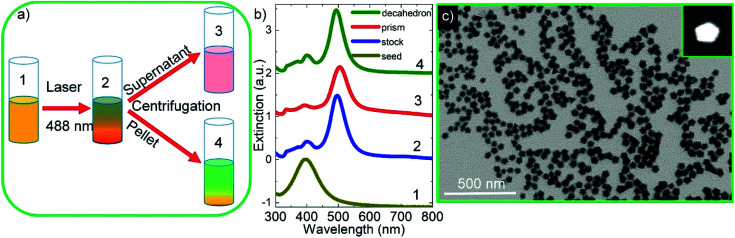
Decahedral silver nanoparticles. (a) Schematic representation of the laser assisted synthesis and separation of nanodecahedra (bicolored) from nanoprisms (monocolored). After the laser assisted synthesis, the colloid contains prism and decahedral shaped NPs. The NPs are selectively separated from each other by centrifugation. (b) Experimental extinction spectra of decahedral, prism, and spherical silver colloids. The peak at around 400 nm in the spectrum is a strong indication of spherical seed silver NPs. (c) STEM image of silver decahedral nanoparticles synthesized by photochemical transformation of spherical silver NPs. The dark field STEM image of silver decahedral NPs is provided in the inset. Pentagon shaped Ag NPs are clearly visible in the dark field image.

**Fig. 3:**
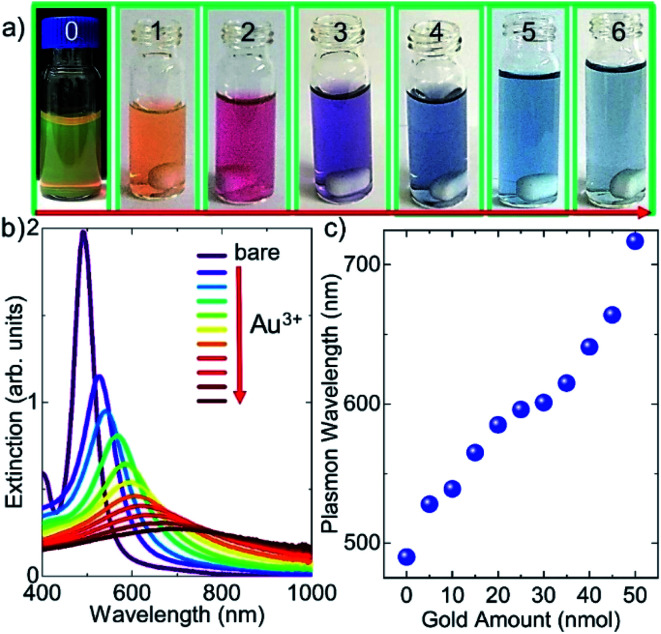
Tuning localized SPP resonance wavelength of the decahedral silver nanoparticles by the galvanic replacement reaction. (a) Photos of the silver nanodecahedral colloid treated with varying amounts of gold ions. The first (0) and second (1) photos (from left to right) indicate the (monometallic) silver decahedral colloid in the dark and bright background, respectively. The other photos having numbers from 2 to 6 represent bimetallic decahedral colloid. The amounts of gold ions introduced into the silver decahedral colloid increase from left to right. (b) Experimental extinction spectra of the decahedral colloid upon addition of varying amounts of gold ions and corresponding localized surface plasmon polariton resonance shift. (c) Tuning localized surface plasmon polariton resonance wavelength with gold ions introduced during the galvanic displacement reaction. The plasmon resonance wavelength can be shifted more than 200 nm.

Fig. 1 shows the schematic representation of photochemical synthesis of decahedral Ag NPs from spherical Ag NPs. Isotropic, spherical Ag NPs synthesized in aqueous medium have localized SPP wavelength at around 400 nm (yellow in color) and their extinction spectra are shown in Fig. 2. By varying the shape of the isotropic Ag nanoparticles, the plasmon resonance wavelength of the nanoparticles can be effectively tuned across the entire visible and near-infrared region of the electromagnetic spectrum. l-Arginine, an amino acid used in the biosynthesis of proteins, was used as a photochemical promoter.^16^ Different from the previous studies where a white light or a blue LED was used as a light source, in this study, a 488 nm blue laser was employed to irradiate the spherical silver NPs, Fig. 2a. In previous studies, lasers were used for the synthesis of monometallic and bimetallic isotropic plasmonic nanoparticles.^28,29^ Extinction spectra of silver NPs obtained after exposing silver seed NPs to the laser showed a sharp peak at around 490 nm, which is the localized SPP resonance wavelength of anisotropic Ag NPs as shown in Fig. 2b. The peak of low intensity at around 400 nm is a very clear indication of seed NPs remaining in the colloid. Note that we have found out by looking at the extinction spectra of the colloid as a function of exposure time that around 15 hour exposure of the colloid is enough to convert nearly all of the seed NPs to anisotropic NPs. Decahedral shaped NPs were selectively separated from prism shaped NPs by centrifugation as shown in Fig. 2a. The decahedral shaped NPs are indeed bicolored but prism shaped NPs are monocolored, see the ESI† for the images. Fig. 2c shows the STEM images of Ag decahedral nanoparticles synthesized by photochemical transformation of aqueous spherical Ag NPs. In addition, the dark field STEM image of Ag decahedral NPs is shown in the inset. The average particle size of the Ag NPs was found to be around 50 nm and they were of decahedral shape. In this study, bimetallic decahedral shaped nanoparticles were synthesized and the plasmon resonance wavelength of the decahedral nanoparticles was tuned by galvanic replacement reactions between Ag and Au^3+^ as demonstrated in Fig. 3. Indeed, in a previous study, the regrowth of the decahedral Ag nanoparticles was achieved by adding spherical Ag nanoparticles to decahedral nanoparticles under visible light exposure and hence monometallic nanoparticles with tunable plasmon resonance wavelength were synthesized.^16^

The galvanic replacement reaction is an electrochemical reaction between silver atoms and gold ions because of difference in their standard electrochemical reduction potentials.Au^3+^ + 3Ag → Au + 3Ag^+^, Δ*E*^o^ = +0.7 V

In fact, the galvanic replacement reaction between silver atoms and gold ions is spontaneous since the free energy of the reaction is negative.^7^Fig. 3a shows photos (from 1 to 6) of the Ag nanodecahedral colloids treated with varying amounts of gold ions. The first and second photos from left to right in Fig. 3a indicate the monometallic Ag decahedral colloid in the dark and bright background, respectively. The other photos having numbers from 2 to 6 represent the bimetallic decahedral colloid. The amounts of gold ions introduced into the Ag decahedral colloid increase from left to right. The extinction spectra of the decahedral nanoparticles upon addition of varying amounts of gold ions and corresponding localized SPP resonance shift are shown in Fig. 3b. Owing to the galvanic replacement reaction between gold ions and silver atoms on the Ag NPs, adding gold ions into the colloid tunes the LSPP resonance wavelength of the colloid and hence the electrochemical process alters the optical properties of the decahedral shaped NPs. Therefore, the localized SPP resonance wavelength of the nanoparticle can be shifted more than 200 nm as shown in Fig. 3c. A gold shell has been coated on the silver decahedral NPs and thus the stability of the silver decahedral nanoparticles has been greatly improved. It should be also noted that during the galvanic replacement reaction a single gold ion replaces three silver atoms, and thus the gold coated NPs may contain very small voids as shown in our previous study.^7^ In fact, the STEM images of the bimetallic decahedral NPs indicate that the shape of the nanoparticles is preserved after the electrochemical displacement reactions, see ESI† for the images. In addition, energy dispersive X-ray (EDX) elemental analysis shows that silver-gold bimetallic decahedral shaped nanocrystals are formed, see the ESI† for EDX images.

**Fig. 4:**
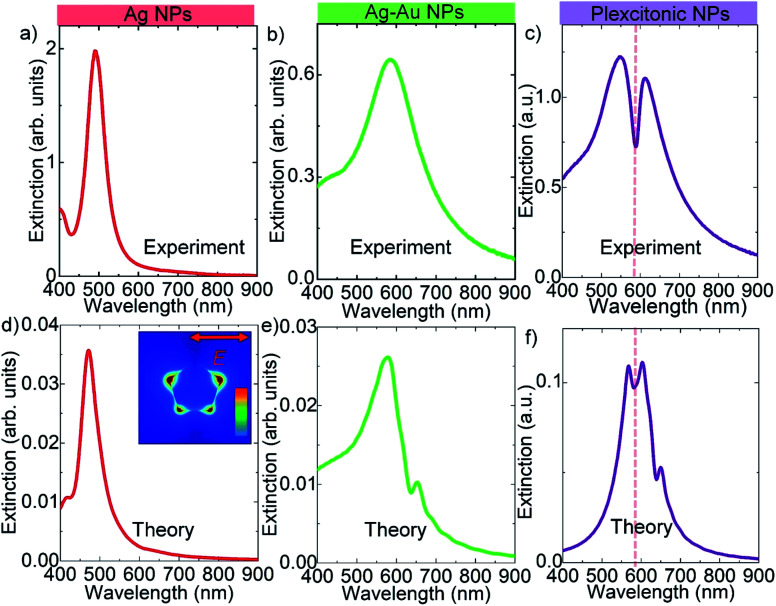
Strong plasmon–exciton coupling in decahedral shaped bimetallic NPs. (a) Extinction spectrum of decahedral Ag (monometallic) nanoparticles in water. Extinction spectra of (b) decahedral Ag–Au (bimetallic) NPs, and (c) decahedral Ag–Au plexcitonic NPs. Theoretically calculated extinction spectra of (d) a decahedral shaped Ag NP, (e) a decahedral shaped Ag–Au NP, and (f) a decahedral shaped Ag–Au plexcitonic NP. The inset in (d) shows the electric field distribution in the decahedral shaped Ag NP in the resonant excitation. The decahedral nanoparticles are uniformly covered with the J-aggregate layer. The splitting in the extinction spectrum at around J-aggregate exciton resonance wavelength is a clear indication of strong coupling between plasmon resonance of decahedral nanoparticles and exciton resonance of the J-aggregate dye.

We now turn our attention to the synthesis of decahedral shaped bimetallic plexcitonic nanoparticles. Decahedral shaped bimetallic plexcitonic nanoparticles were synthesized by self-assembly of the J-aggregate dye on the bimetallic decahedra. The J-aggregate dye used in this work has an exciton resonance wavelength at around 585 nm; see the ESI† for the absorbance spectrum of the J-aggregate dye. However, the decahedral Ag nanoparticles obtained after the photochemical synthesis have plasmon resonance wavelength at around 490 nm, Fig. 4a. In order to spectrally overlap between the plasmon resonance and the exciton transition in J-aggregates (at zero detuning), we colloidally synthesized bimetallic decahedral shaped plasmonic NPs by using galvanic replacement reactions, see Fig. 4b for the extinction spectra. The splitting in the extinction spectrum of the hybrid nanoparticle at around J-aggregate exciton resonance wavelength as shown in Fig. 4c is a clear indication of strong coupling between localized plasmons of decahedral nanoparticles and excitons of the J-aggregate dye. In addition, we theoretically calculated the extinction spectra of a decahedral shaped Ag NP, a decahedral shaped Ag–Au NP, and a decahedral shaped Ag–Au plexcitonic NP, and they are shown in Fig. 4d–f. In the numerical calculations, the decahedral shaped metallic nanoparticle was suspended in the air. The localized Frenkel exciton of the J-aggregate dye was assumed to be Lorentzian and hence expressed as *ε*(*ω*) = *ε*_∞_ + *f*_0_(*ω*_0_^2^/(*ω*_0_^2^ − *ω*^2^ − i*γ*_0_*ω*)) where the resonance wavelength of the oscillator was set to 585 nm (2.11 eV) and the width of the exciton resonance (*γ*_0_) was set to around 32 meV. The background index, *ε*_∞_, was set to 2.1. The oscillator strength of the Lorentz oscillator, *f*_0_, was set to 0 for a bare plasmonic nanoparticle and varied for coupled nanoparticles. A very narrow dip in the extinction spectra in Fig. 4f is indeed a very clear indication of strong coupling between an exciton and a plasmon. It should be pointed out here that Rabi splitting energy, *ℏΩ* ∼ (*f*_0_)^1/2^, depends on the oscillator strength, *f*_0_, of the exciton. The decahedral metallic nanoparticles shown in this work localize electromagnetic waves at sharp corners in a very small region as shown in Fig. 4d. Since the coupling strength (*g*), *ℏΩ* = 2*g* ∼ 1/(*V*)^1/2^, is inversely proportional to the square root of the effective mode volume of the cavity, *V*, we expect to see enhancement in light–matter interaction in the sharp corners of the decahedral shaped plasmonic nanoparticles. Therefore, decahedral shaped plasmonic nanoparticles with very sharp corners can be used to study light–matter interaction in very small region, for example, single molecule strong coupling at room temperature.^6^ Furthermore, by using a coupled oscillator model, the energies of the upper and lower polariton branches of the plexcitons can be represented as *E*_1,2_ = (*E*_p_ + *E*_ex_)/2 ± 1/2((4*g*^2^ + (*E*_p_ − *E*_ex_)^2^))^1/2^ where *E*_1_ and *E*_2_ are the upper and lower polariton energies, *g* is the coupling strength, and *E*_p_ and *E*_ex_ are the plasmon and exciton resonance energies, respectively. At the zero detuning, the energy difference of the polariton branches is called the Rabi splitting energy defined as Δ*E* = *E*_2_ − *E*_1_ = *ℏΩ* = 2*g*. The coupling strength, *g*, is closely related to the local electric field intensity and the number of excitons present in the plasmonic nanoparticle. Including the plasmon damping rate, *γ*_p_, and exciton damping rate, *γ*_ex_, the Rabi splitting is now *ℏΩ* = 4*g*^2^ + (*γ*_p_ − *γ*_ex_)^2^. Strong coupling occurs when the coupling strength is at least larger than (*γ*_p_ − *γ*_ex_)/2. For decahedral shaped plexcitonic nanoparticles demonstrated in this study, a Rabi splitting energy of more than 200 meV can be calculated from Fig. 4c. Owing to the interband transitions in gold, which has an interband edge of around 2.38 eV,^30^ the experimental and theoretical extinction spectra of the bimetallic NPs show a very small transparency dip at around 590 nm in Fig. 4b and e, respectively. In addition, it should be noted that the interband transitions in gold are clearly seen in the extinction spectra shown in Fig. 4b and e when compared with the extinction spectra of the decahedral shaped Ag NPs shown in Fig. 4a and d.

**Fig. 5:**
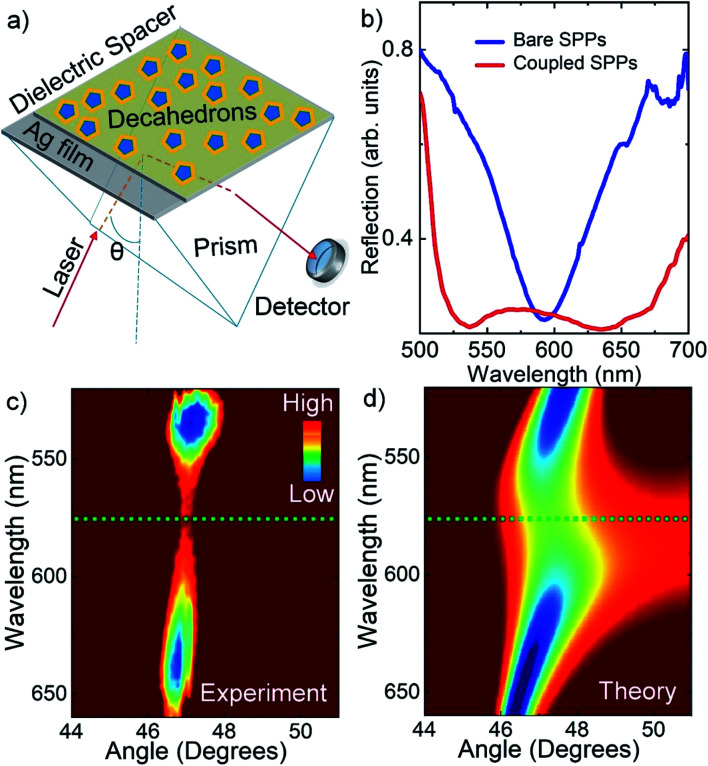
Strong plasmon–plasmon coupling in bimetallic decahedral NPs on a flat silver film. (a) Schematic representation of the experimental set up, Kretschmann configuration, used for the excitation of surface plasmons on the silver film. (b) Reflection spectra of a bare Ag film and a coupled decahedral NPs-Ag film were taken at around 47 degrees. Under the resonance conditions, the reflectivity goes to a minimum value (blue colored region) in the dispersion curves. (c) Experimentally obtained dispersion curve of decahedral NPs on the Ag film. (d) Theoretically obtained dispersion curve of a Lorentz oscillator, *i.e.* representing extinction of bare decahedral nanoparticles, on the Ag film.

We further study plasmon–plasmon coupling in bimetallic decahedral nanoparticles self-assembled on a flat Ag film. Plasmon–plasmon hybridization between localized SPPs of metal nanoparticles and propagating SPPs of flat metal films^27^ can lead to a plasmon resonance shift and enormous electric field enhancements, which are very useful in surface enhanced spectroscopies, strong plasmon–exciton couplings, plasmonic light sources, and solar cells.^31,32^ In order to study plasmon–plasmon coupling in decahedral nanoparticles on flat metal surfaces, the well-known Kretschmann configuration was used.^27^ The Kretschmann configuration is commonly used for the excitation of surface plasmons on thin metal films. Schematic representation of the experimental set up used to investigate coupling of localized and propagating surface plasmon polaritons is shown in Fig. 5a. Polarization dependent spectroscopic reflection measurements were performed for each incidence angle to study coupling between localized and propagating SPPs. The dispersion curves were acquired by using a tunable laser light source with a spectral width of around 1 nm. The excitation of surface plasmons by incident light, surface plasmon polariton formation, is achieved when the horizontal component of the incident light momentum (*k*_x_) is equal to the real part momentum of surface plasmons (*k*_SP_). Under this condition, the dispersion relation is *k*_x_ = *k*_o_*n*_p_ sin(*θ*) = *k*_SP_ = 2π/*λ*(*ε*_m_*ε*_d_/*ε*_m_ + *ε*_d_)^1/2^ where *n*_p_ is the refractive index of the prism, *θ* is the angle of incident light, *λ* is the wavelength of incident light, and *ε*_m_ and *ε*_d_ are the dielectric constants of metal and dielectric, respectively. The flat silver film was chemically modified with self-assembled monolayers of thiols, 16-mercaptohexadecanoic acid, and then further chemically modified with self-assembled monolayers of aminosilanes, (3-aminopropyl)trimethoxysilane. As a result, decahedral NPs in water were electrostatically self-assembled on chemically modified surfaces.^27^ The reflection spectra of a bare Ag film and coupled decahedral NPs-Ag film are shown in Fig. 5b. The corresponding dispersion curve for nanoparticles coupled to Ag film is shown in Fig. 5c. Noted that a bare Ag film showed a dispersion without an anticrossing behavior, see also the ESI.† In fact, an anticrossing behavior in the dispersion curve indicates that the localized SPPs of decahedral NPs strongly couple with the propagating SPPs of the Ag film. The plasmonic nanocavity formed between the decahedral nanoparticles and metal film has strong gap-dependent plasmon resonance.^31^ Resonance wavelength of the nanocavity shifts to longer wavelengths with respect to the resonance wavelength of the bare metal nanoparticle when the gap distance is decreased.^32^ The bare decahedral bimetallic nanoparticles used in Fig. 5c had plasmon resonance at around 550 nm. The plasmon resonance of the nanocavity is at around 575 nm and thus around 25 nm shift in the plasmon resonance wavelength can be calculated. In our previous study, the gap distance between a thin Ag film and Ag nanoprisms was more than 10 nm and therefore there was not large difference between the plasmon resonance wavelength of the bare nanoparticles and the coupled nanoparticles.^27^ The experimental results shown in Fig. 5c were fully supported by theoretical calculations as shown in Fig. 5d. In our theoretical calculations, we assume that the plasmonic nanoparticle is a Lorentz oscillator having a resonance energy of 2.14 eV and a resonance width of 122 meV, see the ESI† for details. Indeed, the plasmonic nanocavity reported in this study can be used to study light–matter interaction in very small region (around 50 × 50 × 5 nm^3^).^6^

## Conclusions

In summary, we have demonstrated laser-assisted synthesis of anisotropic noble metal nanocrystals and colloidal synthesis of bimetallic decahedral shaped plexcitonic nanocrystals with tunable optical properties in the visible region. Decahedral silver nanocrystals were photochemically synthesized from spherical silver nanoparticles by using a 488 nm laser. After the laser assisted synthesis, the monocolored prism shaped silver nanoparticles were selectively removed from the bicolored decahedral silver nanoparticles by centrifugation. In addition, the optical properties of decahedral nanoparticles were tuned by the galvanic replacement reaction between gold ions and silver atoms. In the strong coupling regime, excitons of the J-aggregate dyes and SPPs of decahedral bimetallic nanoparticles couple and hence decahedral shaped plexcitonic nanoparticles are formed. Furthermore, we have shown that localized SPPs of decahedral shaped bimetallic nanoparticles interact strongly with the propagating SPPs of the flat silver film and thus new hybrid polaritonic modes have been generated. The new plexcitonic nanoparticles with very sharp corners localize electromagnetic field in very small region. The decahedral shaped plexcitonic nanoparticles with improved and tunable optical properties in the visible spectrum can be used for understanding light–matter interaction at nanoscale dimensions.

## Conflicts of interest

There are no conflicts to declare.

## Supplementary Material

NA-003-D0NA00829J-s001
