# Supplementary material for: Laser assisted synthesis of anisotropic metal nanocrystals and strong light-matter coupling in decahedral bimetallic nanocrystals
Source: Nanoscale Adv. 2021 Jan 19;3(6):1674–81. doi: 10.1039/d0na00829j (PMC9418786; doi:10.1039/d0na00829j)
Supplement: NA-003-D0NA00829J-s001 [file NA-003-D0NA00829J-s001.pdf]

Electronic Supplementary Material (ESI) for Nanoscale Advances.
This journal is © The Royal Society of Chemistry 2021

Supporting Information for

Laser Assisted Synthesis of Anisotropic Metal Nanocrystals and Strong Light-Matter Coupling in Decahedral Bimetallic Nanocrystals

Fadime Mert Balci,[a] Sema Sarisozen,[b] Nahit Polat,[a] C. Meric Guvenc,[c] Ugur Karadeniz,[d] Ayhan Tertemiz,[a] and Sinan Balci[a*]

[a]Department of Photonics, Izmir Institute of Technology, 35430 Izmir, Turkey

[b]Department of Chemistry, Izmir Institute of Technology, 35430 Izmir, Turkey

[c]Department of Materials Science and Engineering, Izmir Institute of Technology, 35430 Izmir, Turkey

[d]Department of Physics, Izmir Institute of Technology, 35430 Izmir, Turkey

[*]E-mail: sinanbalci@iyte.edu.tr

Table of Contents:

Methods. Synthesis of colloidal nanoparticles and numerical calculations.

Fig. S1 STEM image of silver nanodecahedrons.

Fig. S2 Numerical calculation of a nanodecahedralsilver nanoparticle

Fig. S3 Strong plasmon-exciton coupling in decahedral nanoparticles.

Fig. S4 Extinction spectra of seed and decahedral silver nanoparticle colloids.

Fig. S5 Silver nanodisk and nanodecahedron colloids.

Fig. S6 Schematic representation of the experimental set up.

Fig. S7 Extinction spectra of silver nanoparticles before and after the centrifugation.

Fig. S8 Imaginary and real parts of the dielectric function of Lorentz Oscillator.

Fig. S9 Strong plasmon-plasmon coupling in decahedral Ag NPs on a flat silver film.

Fig. S10. Schematic representation of laser assisted synthesis of decahedral silver NPs.
Fig. S11. EDX elemental analysis results of silver-gold decahedral NPs.
Fig. S12. STEM image of bimetallic nanodecahedrons.
Fig. S13. The composition decahedral plexcitonic nanoparticles.
Fig. S13. FDTD simulation of decahedral shaped plexcitonic nanoparticles.
References

Methods: Synthesis of colloidal nanoparticles and numerical calculations

Chemicals. L-arginine, Polyvinylpyrrolidone (PVP) (40000), $NaBH_4$, $AgNO_3$, trisodium citrate, $HAuCl_4$, were all purchased from Sigma Aldrich and used without any further purification. (5,5′,6,6′-tetrachlorodi(4-sulfobutyl)benzimidazolocarbocyanine (TDBC) dye showing J-aggregate properties at high concentration in aqueous medium was purchased from FEW Chemicals GmbH. Milli-Q water with a resistivity of 18.2 MΩ cm was used in all of the experiments.

Synthesis of Ag seeds and laser assisted synthesis of decahedral nanoparticles. Spherical Ag NPs were used as seeds in the laser assisted synthesis of anisotropic decahedral Ag NPs, see Figure 1 for schematic representation of the laser assisted synthesis of nanocrystals. 0.5 ml of 50 mM sodium citrate was loaded in a glass vial containing 7 ml of water and, subsequently, 15 µl of 50 mM PVP was added. As a source of silver, 200 µl of 5 mM $AgNO_3$ was added. Silver ions were reduced by adding 80 µl of 100 mM $NaBH_4$, a strong reducing agent. The spherical Ag NPs were synthesized by stirring the aqueous solution for several minutes. In fact, the appearance of bright yellow colored solution is a very strong indication of Ag nanoparticle formation. These nanoparticles show localized SPP resonance wavelength at around 400 nm. In the final step, 50 µl of 5 mM L-arginine was quickly injected into this solution combination before the laser irradiation of the spherical Ag colloid. In order to synthesize decahedral shaped

Ag NPs, bright yellow colored Ag colloid was exposed to a 488 nm wavelength blue laser (50 mW, CrystaLaser) for several hours, ~15 hours. It is noteworthy that the shape transformation of the colloid was performed under vigorous stirring at room temperature. In addition, after the synthesis was complete, the colloid was centrifuged at 5000 rpm for 15 minutes in order to remove unreacted reaction precursors, and reaction byproducts.

Synthesis of Ag-Au alloyed nanoparticles. In order to synthesize bimetallic decahedral NPs, firstly, the Ag decahedral colloid was synthesized, Figure 1. After the reaction, 4 ml of the synthesized colloid was centrifuged two times at 5000 rpm for 15 minutes and then the pellet was dispersed in 1 ml water. Afterwards, 100 µl of 50 mM trisodium citrate was added and 50 µl of 0.05 mM $HAuCl_4$ solution was then added dropwise into the Ag nanodecahedral colloid for several times for obtaining Ag-Au alloyed NPs. Owing to the galvanic exchange of silver atoms with gold ions, the silver decahedral nanoparticles were coated with gold. Therefore, the stability of bimetallic nanoparticles is significantly improved as compared to the monometallic Ag nanoparticles due to the stability of gold. Increasing the total amount of $HAuCl_4$ injected into the Ag decahedral colloid resulted in coating of decahedral nanoparticles. Further, Ag-Au alloyed nanoparticles were centrifuged at 15000 rpm for 10 minutes. After the centrifugation was complete, the supernatant was discarded, and the precipitate was then redispersed in water.

Synthesis of plexcitonic decahedral NPs. Plexcitonic decahedral shaped NPs were prepared by mixing 1 ml of the alloyed decahedral colloid with varying amount of 0.1 mM TDBC dye, see Figure 1. The mixture was overnight incubated in the dark. Thereafter, the obtained colloid was centrifuged at 15000 rpm for 10 minutes. In order to remove all of the uncoupled dye molecules from the colloid, the supernatant was discarded, and the precipitate was dispersed in water again. Therefore, the decahedral shaped plexcitonic NPs shown in this study do not contain any uncoupled dye molecules or bare plasmonic nanoparticles in the colloid.

Characterization. In order to investigate morphological evolution of the nanocrystals, Scanning Transmission Electron Microscopy (STEM) (SEM; Quanta 250, FEI, Hillsboro, OR, USA) was used. The samples were prepared by drop-casting nanocrystal suspensions onto 200 mesh carbon-coated copper grid. Extinction measurements of the colloids in a 1 cm quartz cuvette were performed by using a balanced deuterium−tungsten halogen light source (DH2000-BAL, Ocean Optics), and a fiber coupled spectrometer (USB4000, Ocean Optics). All of the characterization measurements were carried out at room temperature.

Numerical calculations. The finite difference time domain (FDTD) method was applied to investigate optical properties of bare and coupled plasmonic nanoparticles, and plasmon-plasmon hybridization in decahedral nanoparticles on flat Ag films. The size of the decahedral shaped nanoparticles was indeed deduced from the STEM images. In FDTD simulations, the plane wave moves in the z-axis. The mesh size is 1 nm during the extinction spectra simulations and 0.1 nm during the electric field map simulations. The electric field polarization is varied and the resulting electric field distribution for each electric field polarization is then obtained in a heat map, see Supporting Information. In the numerical calculations, the decahedral shaped metallic nanoparticle was suspended in the air. The localized Frenkel exciton of J-aggregates was assumed to be a Lorentzian lineshape. A very narrow dip in the extinction spectra is indeed a very clear indication of strong coupling between an exciton and a plasmon. FDTD simulation of plasmon-plasmon coupling was investigated in the Kretschmann configuration. A prism was used to couple incident light to surface plasmons of metal film. The localized SPPs of decahedral nanoparticles were assumed to be Lorentzian and expressed as $\varepsilon(\omega)=\varepsilon_\infty+f_0(\omega_0^2/(\omega_0^2-\omega^2-i\gamma_0\omega))$ where the resonance wavelength of the oscillator was set to 579 nm (2.14 eV), the width of the plasmon resonance (γ_0) was set to around 122 meV. The background index, ε_∞, was set to 2.1. Theoretical dispersion curves were obtained by acquiring the reflection spectra

for each incidence angle within a broad wavelength range and then the resulting reflectivity distribution for each incidence angle was obtained in a heat map, see Supporting Information.

Plasmon-plasmon coupling. In order to study plasmon-plasmon coupling in decahedral nanoparticles on flat metal surfaces, well-known Kretschmann configuration was used.[1] The Kretschmann configuration is commonly used for the excitation of surface plasmons on thin metal films.[2] Flat silver films on a glass substrate was grown by thermal evaporation of Ag under vacuum. The thickness of the Ag film is 40 nm and the purity of the Ag is 99.9%. Glass substrates were cleaned with a piranha solution, a 3:1 mixture of sulfuric acid (95%) with hydrogen peroxide (30%). The dispersion curves were generated by using a tunable laser light source with a spectral width of around 1 nm; i.e., supercontinuum laser (Koheras-SuperK Versa) with acousto-optic tunable filter working in the visible and near infrared region of the electromagnetic spectrum. A glass prism made of BK7 was used to couple incident light to surface plasmons on flat Ag film. An index matching fluid was used to provide the index continuity between the glass substrate and the right angle glass prism. Therefore, surface plasmon of flat silver film and photons of the incident light strongly couple and hence surface plasmon polaritons are generated on the flat metal films. Freshly prepared Ag thin films were dipped into 10 ml of 10 mM 16-mercaptohexadecanoic acid (90%) in isopropanol for 30 minutes and then the substrates were washed with ample amount of isopropanol. Afterwards, the surface was further modified with 30 mM (3-aminopropyl)trimethoxysilane (97%) in isopropanol for 30 minutes and then the substrates were washed with ample amount of isopropanol and then water. Finally, decahedral bimetallic colloid was placed on top of the modified Ag film for 30 minutes and then washed with ample amount of water. Subsequently, the coupled decahedral bimetallic nanoparticle-film structure was used for optical characterization.

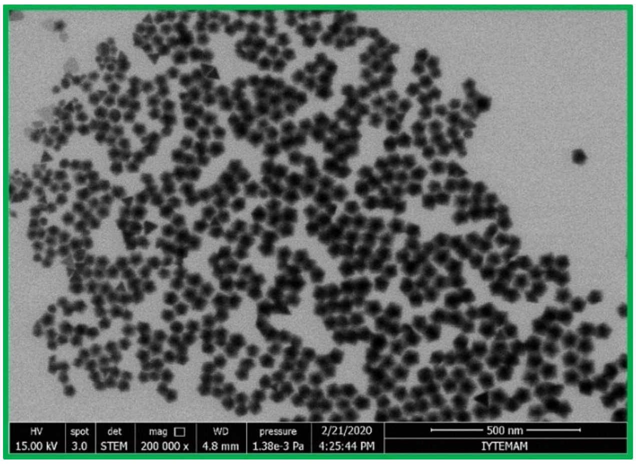

Figure S1. Plasmonic silver nanodecahedrons. Large area STEM image of nanodecahedrons synthesized by photocatalytic reaction. Silver seed nanoparticles are converted to silver nanodecahedral nanoparticles by using a laser light at 488 nm in the presence of L-arginine. Most of the silver nanoparticles are decahedron in shape.

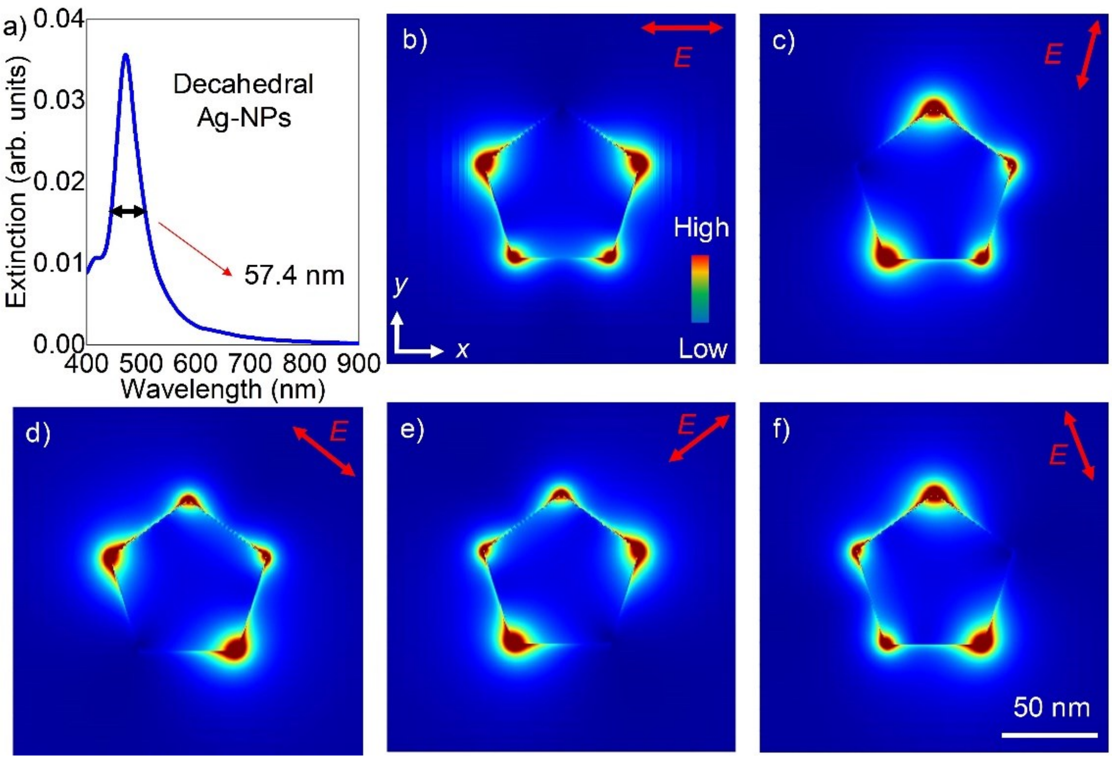

Figure S2. Numerical calculation of a nanodecahedral silver nanoparticle. (a) Theoretically calculated extinction spectrum of a single decahedral Ag NP having localized surface plasmon polariton resonance at around 472 nm. The full width at half maximum of very sharp plasmon resonance is around 57.4 nm. (b-f) Electric field distribution of a single silver nanodecahedron at resonance wavelength with varying electric field polarization. The red arrow in each graph indicates the polarization of the electric field. Electric field is localized at sharp corners.

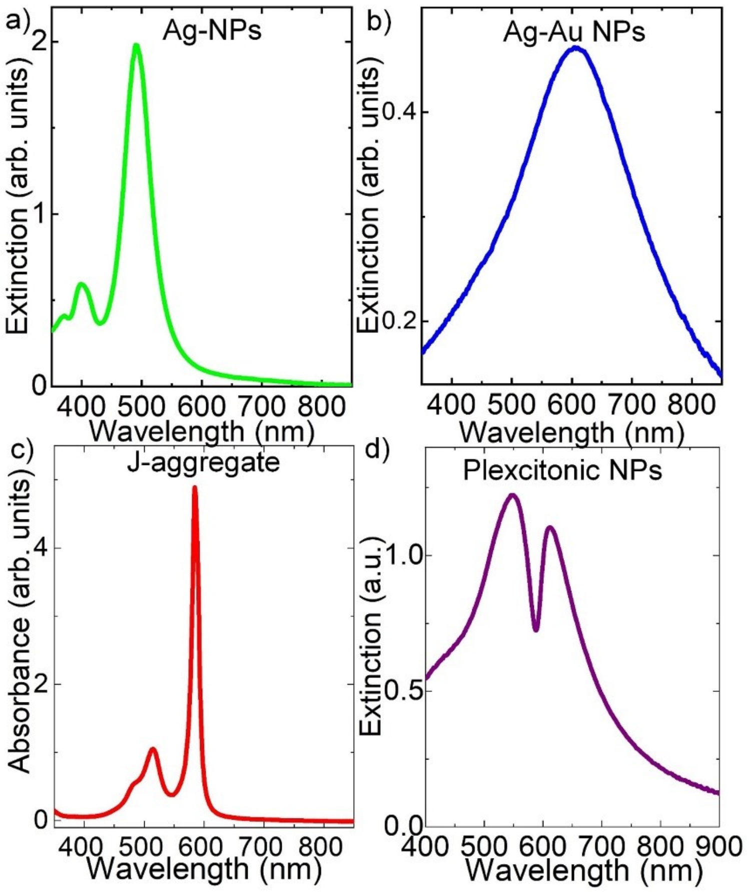

Figure S3. Strong plasmon-exciton coupling in decahedral shaped Ag-Au NPs. (a) Extinction spectrum of decahedral silver nanoparticles. (b) Extinction spectrum of decahedral Ag-Au NPs in water. (c) Absorbance spectrum of J-aggregate dyes in water. The broad absorbance peak at around 510 nm belongs to dye molecule mononers and the sharp peak absorbance peak at around 585 nm belongs to aggregated dye molecules, J-aggregates. (d) Extinction spectrum of decahedral Ag-Au plexcitonic NPs.

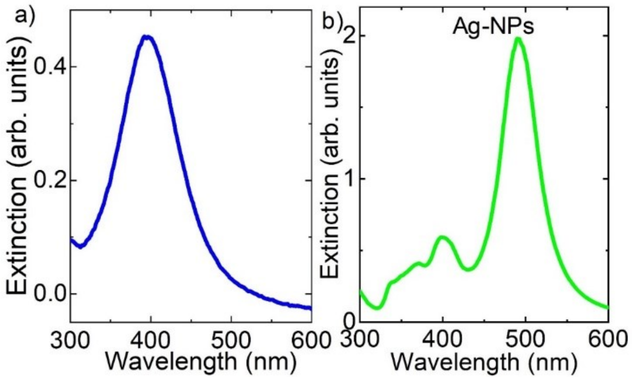

Figure S4. (a) Extinction spectrum of seed silver nanoparticle colloid. The spherical seed nanoparticles, possessing isotropic shape, have localized surface plasmon polariton resonance wavelength at around 400 nm. The spherical silver seed nanoparticle colloid is yellow colored. (b) Extinction spectrum of decahedral silver nanoparticles obtained after exposing silver seed nanoparticles with a laser, 488 nm. The sharp peak at around 490 nm is localized surface plasmon polariton resonance of decahedral shaped silver nanoparticles. The peak of low intensity at around 400 nm is indication of seed nanoparticles in decahedral nanoparticle colloid.

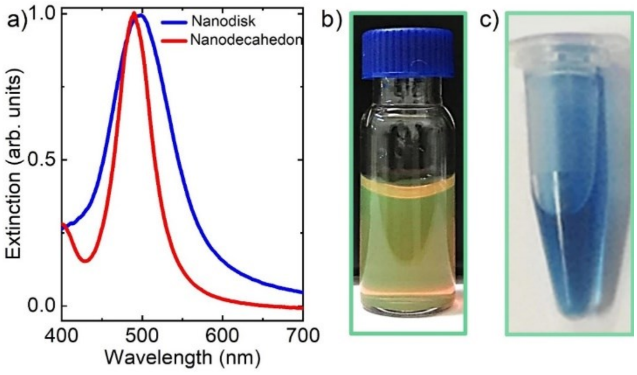

Figure S5. (a) Extinction spectra of the silver nanodisk and nanodecahedron colloids. The decahedral shaped silver nanoparticles have sharper localized surface plasmon resonance peak than nanodisk shaped silver nanoparticles. (b) A photo of bicolored silver nanodecahedron colloid. (c) A photo of monocolored silver nanodisk colloid.

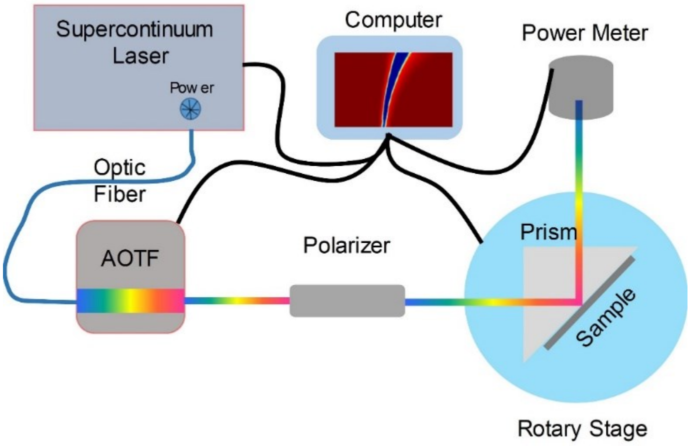

Figure S6. Schematic representation of the experimental set up used to obtain coupling of localized and propagating surface plasmon polaritons in the visible spectrum. Polarization dependent spectroscopic reflection measurements were taken to study coupling between localized and propagating surface plasmon polaritons. The dispersion curves were generated by using a tunable laser light source with a spectral width of around 1 nm; i.e., supercontinuum laser (Koheras-SuperK Versa) with acousto-optic tunable filter working in the visible and near infrared.

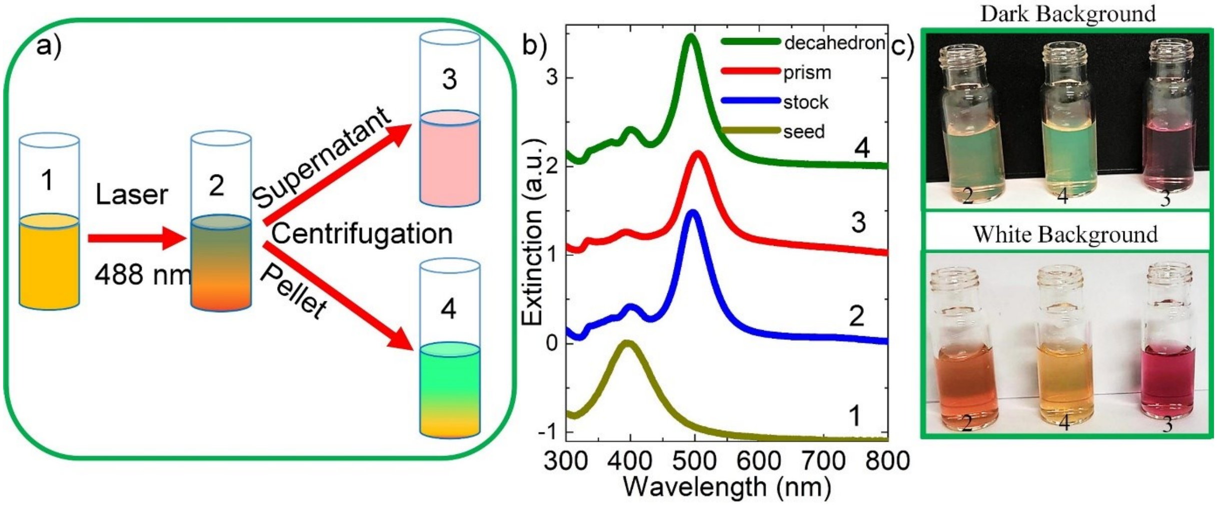

Figure S7. Decahedral shaped silver nanoparticles before and after the centrifugation. (a) Schematic representation of laser asisted synthesis of decahedral NPs and separation of decahedral NPs from prism NPs. (b) Extinction spectra of decahedral, spherical, prism shaped NPs. (c) Photos of bicolored and monocolored silver NPs.

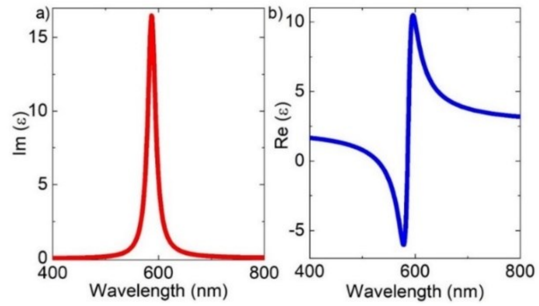

Figure S8. (a) Imaginary (b) Real parts of the dielectric function of Lorentz Oscillator used for modeling J-aggregate thin film on decahedral silver-gold bimetallic nanoparticles in FDTD simulations.

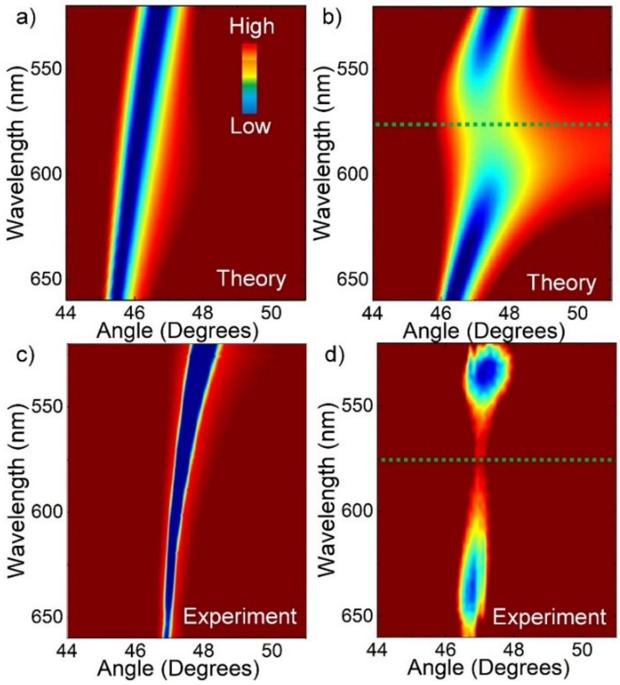

Figure S9. Strong plasmon-plasmon coupling in decahedral Ag NPs on a flat silver film. (a) FDTD simulation of dispersion curve of bare Ag film. (b) FDTD simulation of dispersion curve of Ag film coated with a Lorentz oscillator. (c) Experimental dispersion curve of bare Ag film. (d) Experimental dispersion curve of decahedral Ag NPs self-assembled on Ag film. The red and blue colors indicate high and low reflectivity in the dispersion curves, respectively. At the resonance condition, the reflectivity goes to a minimum value (blue colored region) in the dispersion curves.

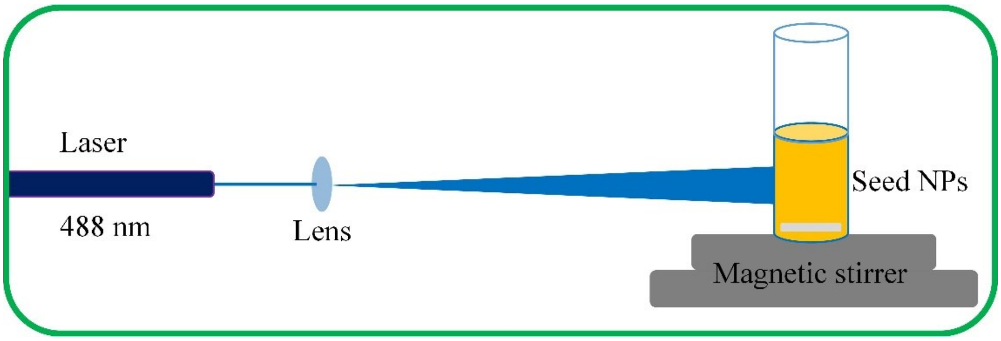

Figure S10. Schematic representation of laser assisted synthesis of decahedral silver NPs from spherical silver NPs. A 488 nm laser was used during the synthesis. The laser beam was expanded by using a lens. The synthesis was performed at room temperature. The colloid was stirred during the synthesis.

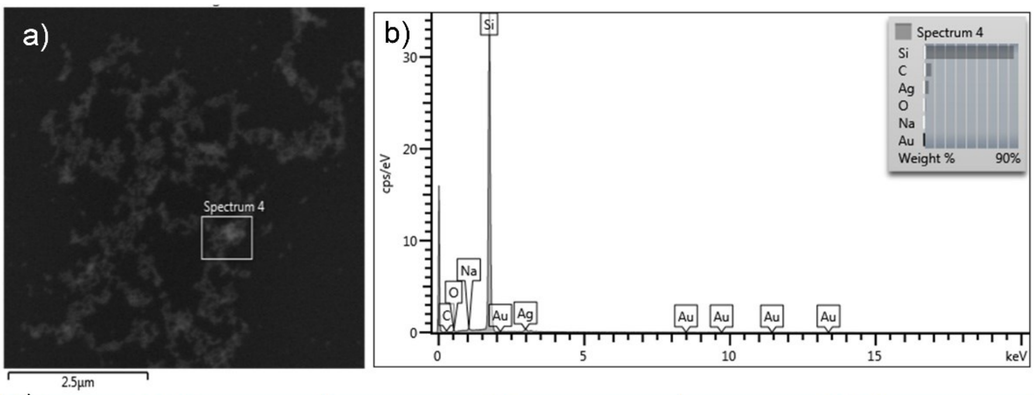

c)

Element	Wt%	Atomic %
C	7.18	15.62
O	1.85	3.02
Na	0.45	0.51
Si	85.57	79.67
Ag	4.75	1.15
Au	0.21	0.03
Total:	100.00	100.00

Figure S11. EDX elemental analysis results of silver-gold decahedral NPs. (a) Large area SEM image of the bimetallic NPs. (b) EDX spectrum of the enclosed area indicated in (a). (c) Elemental composition from the EDX analysis. The table shows the elements detected by EDX analysis from gold-silver decahedral nanoparticles. The bimetallic nanoparticles were placed on a silicon substrate and therefore silicon element also exist in the spectrum shown in (b).

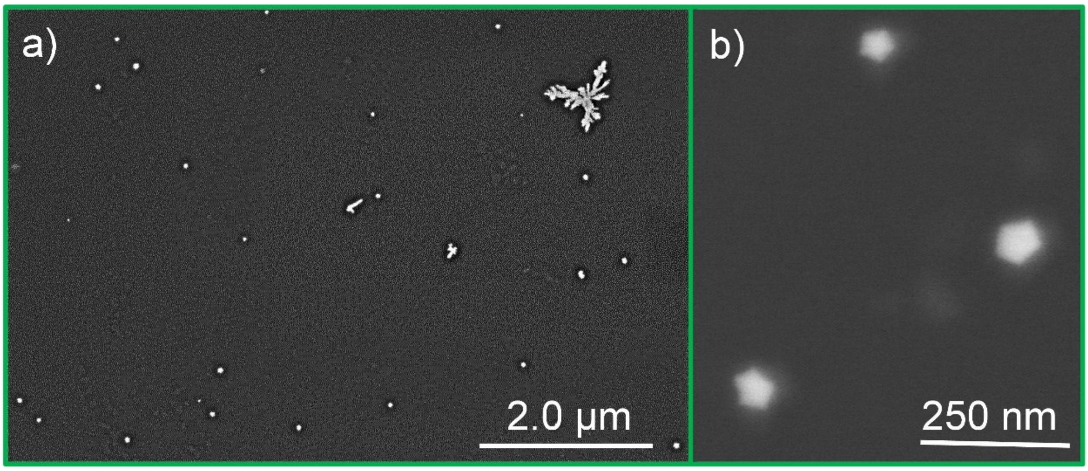

Figure S12. STEM image of bimetallic nanodecahedrons. (a) Large area STEM image of bimetallic nanodecahedrons. After the laser asisted synthesis of silver nanodecahedrons, the silver nanoparticles were treated with varying amount of gold ions. The STEM images show that the shape of the nanoparticles are indeed decahedrons.

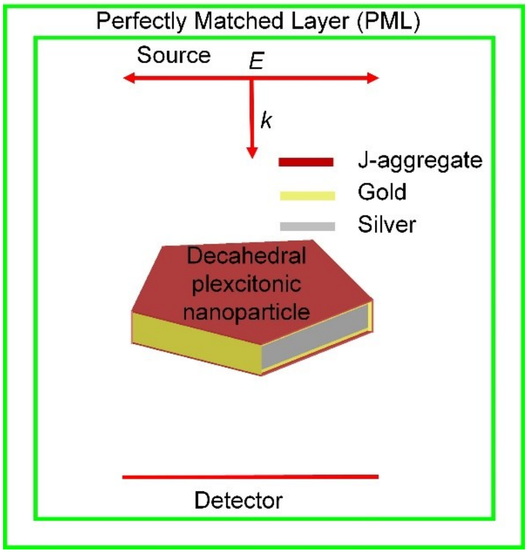

Figure S13. FDTD simulation of decahedral shaped plexcitonic nanoparticles. The decahedral plexcitonic nanoparticle consists of silver core covered with gold layer and J-aggregate layer. The oscilator strength of the J-aggregate layer determines the magnitude of the Rabi splitting energy.

References

1. Balci, S.; Karademir, E.; Kocabas, C., Strong coupling between localized and propagating plasmon polaritons. *Opt Lett* **2015,** *40* (13), 3177-3180.
2. Pockrand, I.; Brillante, A.; Mobius, D., Exciton Surface-Plasmon Coupling - an Experimental Investigation. *J Chem Phys* **1982,** *77* (12), 6289-6295.
